# Scaling up of electronic case-based disease surveillance reporting for COVID-19 and other notifiable diseases through capacity building and antigen RDT provision: a case study of Sierra Leone

**DOI:** 10.3389/fpubh.2025.1574116

**Published:** 2025-05-15

**Authors:** James Sylvester Squire, Ian Njeru, Robert Musoke, Joseph Sam Kanu, Fatmata Bangura, Zikan Koroma, Doris Harding, Stephen Sesay, Bridget Magoba, Ismail Mahat Bashir, Victor Caulker, Mohamed Vandi, Innocent Bright Nuwagira, Charles Njuguna

**Affiliations:** ^1^Ministry of Health, Freetown, Sierra Leone; ^2^World Health Organization, Freetown, Sierra Leone; ^3^District Health Management Team, Western Area Urban, Sierra Leone; ^4^African Field Epidemiology Network, Freetown, Sierra Leone; ^5^World Health Organization - Regional Office for Africa, Brazzaville, Republic of Congo

**Keywords:** electronic, case-based, disease surveillance, COVID-19, Sierra Leone

## Abstract

**Introduction:**

Electronic case-based disease surveillance (eCBDS) provides timely and detailed data collection on diseases and their risk factors for control actions. Use of eCBDS is still low in many African countries including Sierra Leone due to technological, financial, and human resource challenges. Sierra Leone started using eCBDS in 2019 and the COVID-19 pandemic provided the right opportunity for scale up. To support the scale up, a capacity building project was carried out on use of eCBDS for COVID-19 reporting as well as use of COVID-19 antigen rapid diagnostic kits (RDTs). This paper describes how the capacity building was conducted and the outcomes.

**Methods:**

This was a descriptive study where 607 health workers from 155 health facilities in 16 districts were trained on COVID-19 case-based reporting and RDTs use. The training was conducted in phases from November 2021 to June 2022 and post-training monitoring for impact was done up to May 2024. Data collection was done mainly through the eCBDS system where quantitative data was downloaded and analyzed for response timelines. Qualitative data was collected from key informants from selected health facilities using a semi-structured questionnaire.

**Results:**

The number of health facilities that had ever reported a case of a notifiable disease through the eCBDS in the country was 385/1423 (27%) as of 30th June 2021 (before training) and this increased to 58% as of 30th May 2024 (endline). The total number of cases (all diseases) reported in eCBDS from January 2019 to 30th May 2024 was 54,794. Of the reported cases, 44,908 (82%) were suspected COVID-19 cases of which 7,634 (17%) were confirmed positive. Before the training, 97.3% of suspected COVID-19 cases were notified to the district by the health facilities within 24 h, and this improved slightly to 98.1% afterwards. Case investigation with sample collection within 24 h of notification improved from 91.6 to 98.2% before and after the training, respectively.

**Conclusion:**

The COVID-19 pandemic provided a unique opportunity for the country to scale up eCBDS in more health facilities, and this improved notification and investigation timelines. However, more still needs to be done to ensure countrywide use of eCBDS.

## 1 Introduction

**Figure 1:**
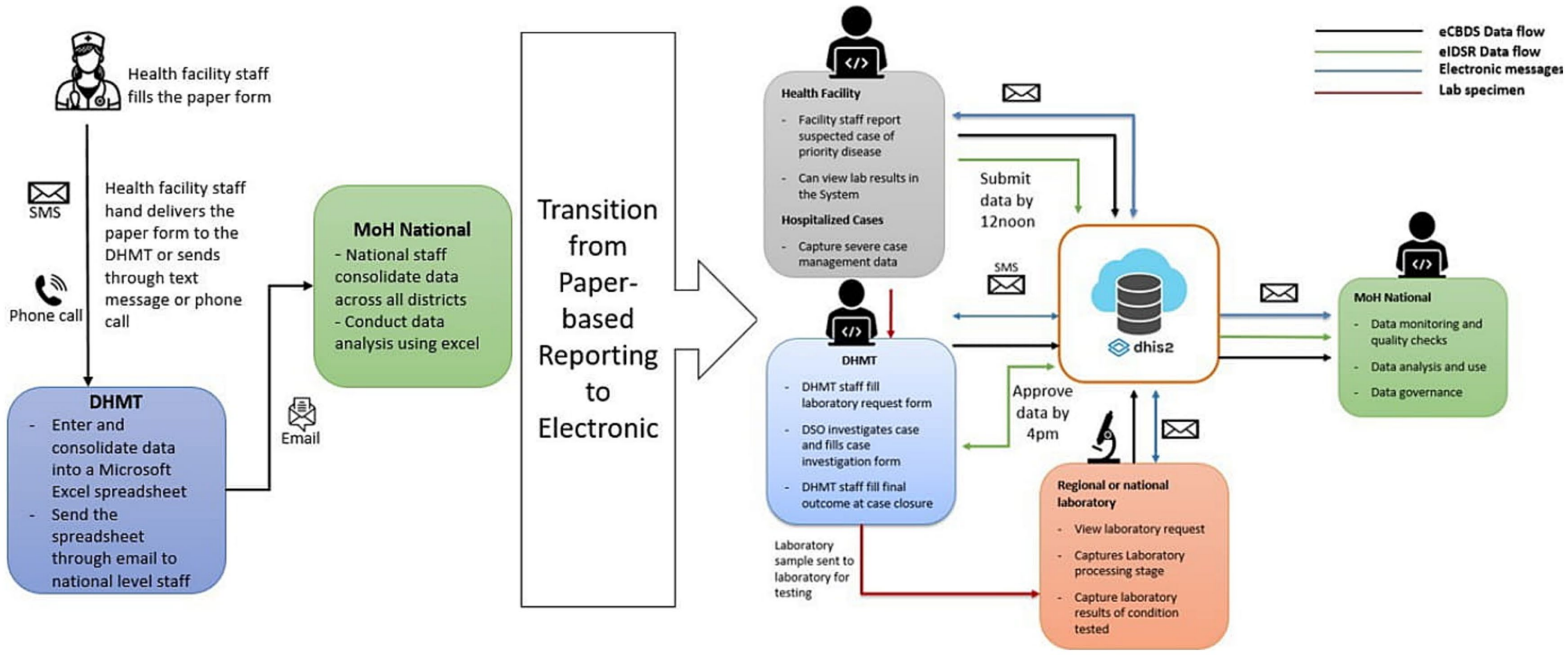
Diagram showing change in data collection from paper-based to electronic reporting in Sierra Leone.

Sierra Leone implements public health disease surveillance through the Integrated Disease Surveillance and Response (IDSR) strategy that was introduced by the World Health Organization (WHO) Regional Office for Africa in 1998 (1). By the end of 2017, 44 of 47 (94%) WHO AFRO member states were already implementing IDSR (2). Sierra Leone officially adopted the IDSR strategy in 2008, although this was not fully implemented until 2015 when the country revitalized the surveillance system following the West Africa Ebola outbreak of 2013–2016 (3). Before the outbreak, both surveillance data collection and reporting were exclusively paper-based. Similarly, the surveillance reporting system consisted of health facilities making phone calls, sending text messages and hand-delivering both aggregated and case-based reporting forms to the District Health Management Teams (DHMT) level on a weekly or expedient basis, respectively. The DHMT staff would then enter the data into a Microsoft Excel spreadsheet, which is emailed to the national level. The surveillance system by then was characterized by poor quality data, untimely reporting, incomplete and inaccurate data. However, as the country embarked on revitalizing its surveillance system to address gaps that may have led to delays in the detection of Ebola and other notifiable diseases, an electronic reporting of aggregated surveillance data using what is called electronic IDSR (eIDSR) was adopted. The eIDSR was built on the District Health Information System 2 (DHIS2) platform, which was adopted by the country as the official health management information system (HMIS) for routine monthly reporting in 2008 (4). DHIS2 is a sustainable open-source platform that has been widely adopted by more than 80 countries globally, including most of the African member states (5). See Figure 1 showing the transition from paper-based to electronic surveillance data reporting.

The eIDSR has been successfully implemented in Sierra Leone with aggregated reporting of surveillance data being done by over 1,500 health facilities in the country. In fact, Sierra Leone was the first country in Africa to have a nationwide electronic health facility reporting of surveillance data which was achieved in 2019 (6). The average investment to enable for electronic reporting was about $1,000 per health facility (7). This success was demonstrated by marked improvement in completeness of IDSR health facility reporting from 84.5% in 2016 to 96% in 2021 and timeliness of IDSR reports from 80.3 to 92% in 2021 (8). As of June 2024, the average completeness of health facility reporting for eIDSR was more than 99% while timeliness was more than 95%. The good reporting rates are beneficial for the country as electronic reporting of integrated disease surveillance and response has been shown to have several benefits including timely reporting and response to alerts and disease outbreaks (9).

While eIDSR was a great electronic tool in collecting and reporting aggregated data, it was not quite suited for case-based reporting. A good surveillance system for effective outbreak detection and response allows for both aggregate data as well as case-based data reporting (10). Unlike aggregate data reporting, case-based data is important to public health practice as it allows better understanding of a disease and its risk factors and hence guide appropriate actions for control. Case-based data also allows detailed data collection for each case or person with a disease and involves collection of demographics, geographical, clinical and epidemiological information including risk factors (11). In countries where electronic health records (EHR) are largely in use, case-based data is usually easily generated from the systems and has the potential to further increase the breadth, detail, timeliness, and completeness of public health surveillance and thereby provide better data to guide public health interventions as well as bridge the gap between public health practice and clinical medicine (12, 13).

In 2017, Sierra Leone leveraged on the success of the electronic reporting of aggregated+ data to develop an electronic case-based disease surveillance (eCBDS) system for reporting individual cases from health facilities to a centralized national data repository (14). The eCBDS system was tested in 4 of 16 districts during 2018–2019 for 20 of the 26 reportable epidemic-prone diseases (4). The eCBDS was built using the tracker in the DHIS2 platform and this provided an opportunity for sustainability as this was an already existing system that was in use. During the rollout of the 3rd edition of IDSR technical guidelines in the county in 2020, at least one health worker from all the health facilities in the country was trained on use of the eCBDS. However, due to inadequate time allocated for the training and the few cases of priority notifiable diseases seen at health facility, the use of eCBDS platform was slow.

In the absence of many notifiable diseases in health facilities, the COVID-19 pandemic provided the right opportunity for scaling up use of eCBDS in the country. COVID-19 pandemic was declared as a Public Health Emergency of International Concern (PHEIC) on 30^th^ January 2020 (15) and a pandemic on 11th March 2020 (16). The first case of COVID-19 was reported in Sierra Leone on 31st March 2020 and the disease was therefore added as one of the reportable priority diseases in eCBDS since the system had already been configured in February 2020. Reporting by health facilities was possible by leveraging the existing eIDSR and eCBDS infrastructure that included use of smart mobile devices and trained health workers (4).

By 30th June 2021, the eCBDS system had reported about 30,573 suspected COVID-19 cases of which 5,705 (19%) were laboratory confirmed. However, since the eCBDS was started in 2019, only 385/1,423 (27%) reporting health facilities at the time had ever reported a case through the eCBDS. We therefore started a capacity building project in July 2021 to train health workers from all the 16 districts on use of eCBDS to enhance reporting and response for COVID-19 and other notifiable diseases. This paper describes how the capacity building was conducted and the outcomes.

## 2 Materials and methods

### 2.1 Study design

This was a descriptive study where mixed methods were used to assess the impact of the training of health workers on the use of eCBDS for COVID-19 detection, reporting and response. The training period was observed and documented from November 2021 to June 2022. Continued monitoring on the use of the eCBDS system for COVID-19 and other diseases continued after the training up to May 2024.

### 2.2 Study setting

The eCBDS system was configured and COVID-19 added as one of the notifiable medical conditions in February 2020. All suspected cases of COVID-19 from all the 16 districts in the country were therefore supposed to be reported through the system. However, most of the health facilities had not been trained on how to report cases through the eCBDS. Therefore, District Surveillance Officers who were in-charge of case investigations were carrying out most of the case-based reporting through the eCBDS.

A review of the eCBDS data as of June 2021 showed that use of the eCBDS had not taken off in many health facilities and only 385/1,423 (27%) reporting health facilities had ever reported using the system since 2019. To enhance the capacity of more health facilities to use eCBDS to report and respond to COVID-19 and other notifiable diseases, a capacity building project was started in July 2021 to train health workers from all the 16 districts on use of eCBDS. The training focused on the use of antigen rapid diagnostic kits (RDTs) for COVID-19 detection as well as reporting of cases using eCBDS. The expected outcomes were increased health facilities reporting COVID-19 and other notifiable diseases through eCBDS and improved disease notification and response time.

### 2.3 Selection of health facilities and health workers for training

**Figure 2:**
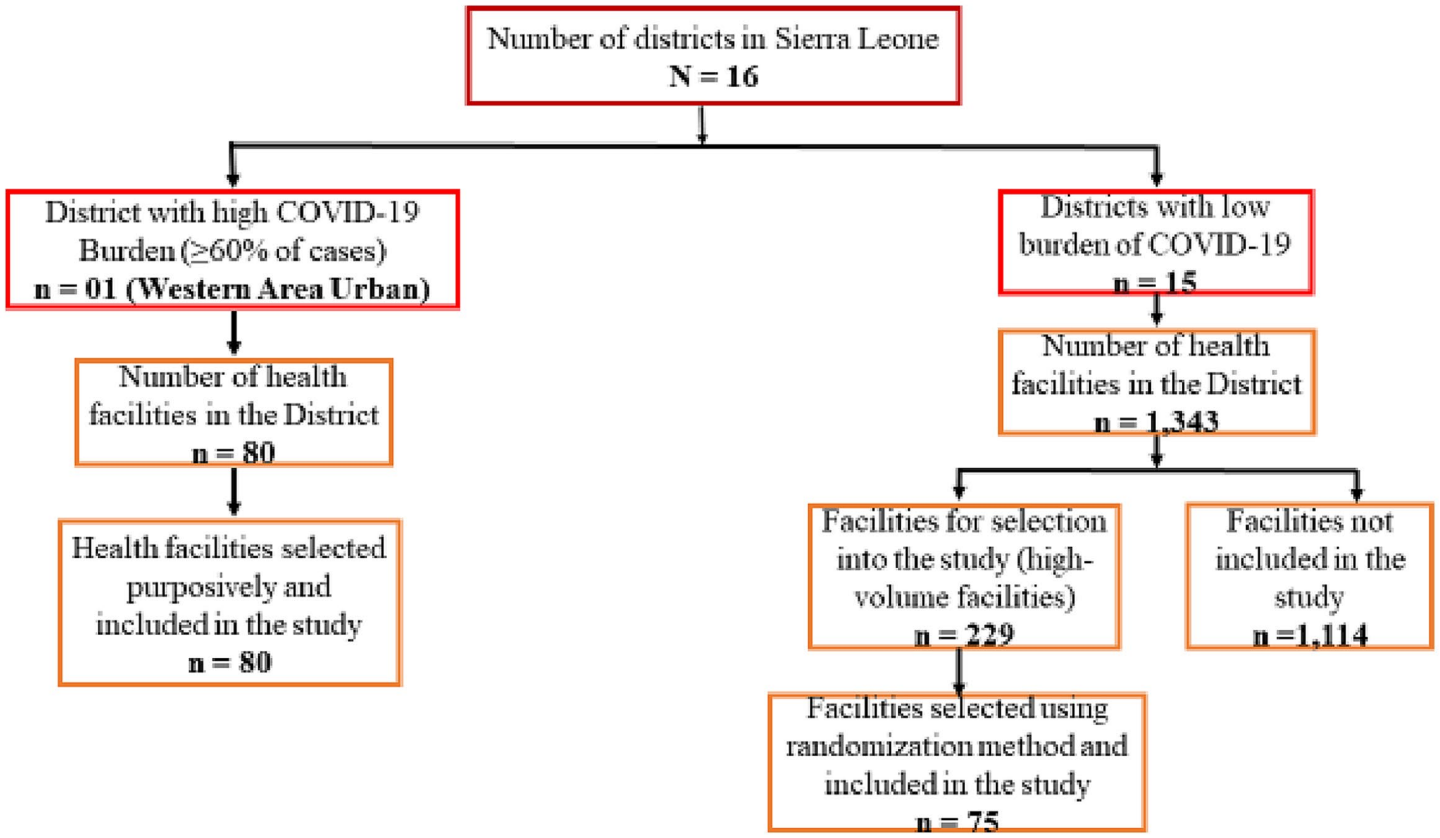
Selection criteria of health facilities included in the study.

Selection of priority health facilities for training was determined by the burden of COVID-19 and the availability of COVID-19 RDTs at the baseline in July 2021. The number of health facilities from the 16 districts in the country at the time was 1,423. However, 60% of the COVID-19 cases reported nationally were from Western Area Urban (WAU) district which is the capital city with about 80 health facilities at the time. Based on the burden of the disease, we purposively selected all 80 health facilities in WAU (Figure 2).

Analysis of the COVID-19 data from the other 15 districts showed that most of the cases were from high-volume facilities that provide outpatient and inpatient services. These facilities are hospitals serving the entire district or community Health Centers (CHCs) serving a population of over 500,000. A total of 229 high-volume facilities were identified in the 15 districts. For the purpose of this study and to ensure all trained facilities are supplied with COVID-19 RDTs, we randomly selected five high-volume health facilities from each of the 15 districts (Figure 2). These facilities were provided with COVID-19 RDTs after the training to ensure that reporting in eCBDS was evidence based.

For each of the selected health facilities, at least three health workers were trained: one clinician, the surveillance focal person and one laboratory technologist. However, up to five clinicians were trained for the district hospitals or equally big hospitals. In total, 607 health workers were trained from 155 health facilities that were drawn from all the 16 districts. The trained personnel were requested to conduct on-job training and mentorship to other health workers in their health facilities.

### 2.4 Training content and process

A two-day training was planned and conducted for the national, district and selected health facility staff. The training covered both theory and practical use of COVID-19 RDTs testing, the use of eCBDS system for reporting and data analysis. Specifically, the health workers were trained on the IDSR standard case definitions including COVID-19, how to fill the IDSR notification form (hard copies and electronic version), submit reports and analyze data in eCBDS, how to collect samples for COVID-19 and how to test samples using rapid diagnostic kits for COVID-19. The two-day training sessions were carried out as follows: day 1 was the opening session, and presentations on the training objectives, an overview of public health surveillance, case definitions of priority disease conditions with a focus on COVID-19 epidemiology, and types of samples to collect for COVID-19. Day 2 covers mainly the practical sessions with ample time given to health staff to practice the use of the eCBDs system to capture, retrieve and analyze data. The practical sessions also included the proper procedure to collect a nasopharyngeal sample for COVID-19 testing, preparation of RDT and interpretation of test results. To address variation in digital literacy among learners, the trainers encouraged peer-to-peer support and spend more time with slow learners. Each of the training days lasted 8 h with tea and lunch breaks in between sessions. The training also included group work, knowledge test and plenaries all geared toward enforcing learning.

The training was cascaded at three levels: first, 10 national trainers of trainers (TOTs) were trained. Second, 5–10 district level TOTs per district were trained by the national TOTs. Third, 3–8 health workers per health facility were trained by a combination of national and district TOTs. The training was conducted in two phases. Phase 1 was conducted in November and December 2021 and a total of 350 health workers were trained from 80 health facilities in Western Area Urban district (Capital City) where 60% of COVID-19 cases in the country were reported from. Phase 2 training was conducted in May and June 2022 and a total of 257 health workers were trained from the remaining 15 districts. The time lag between phase 1 and phase 2 was to allow for full implementation in WAU to draw lessons that could be applied in the remaining 15 districts.

Following the training, the national and district trainers carried out two rounds of structured supportive supervisory visits to the health facilities. A structured checklist was developed and administered during the visit. Data was collected on the usefulness and ease of use of the eCBDS system, the quality of data entered in the eCBDs, compliance of patients and use of the COVID-19 RDTs, requests by clinicians for COVID-19 RDT tests, and knowledge by healthcare workers on the use of COVID-19 RDTs. During the visit, health facility staff were mentored and challenges encountered in using COVID-19 RDTs, as well as reporting through eCBDS, were addressed by the supervision teams.

### 2.5 Laboratory testing for COVID-19

Testing for COVID-19 during the study was done at the health facilities using Panbio antigen RDTs (nasopharyngeal) from Abbott Diagnostics. However, facilities were allowed to use any other testing methods that were available, such as PCR or other types of antigen-based RDTs for COVID-19. All positive cases by antigen RDT were assumed to be confirmed cases of COVID-19 as per the WHO and country COVID-19 guidelines and were therefore reported using eCBDS for follow up by the District Surveillance Officers.

### 2.6 Data reporting process through eCBDS

Reporting of COVID-19 cases was done through the eCBDS system. The health facilities could access the eCBDS system through a DHIS2 Android application on their smart mobile devices.

Initially, all suspected cases of COVID-19 were reported through the eCBDS by health facilities. However, this was associated with a lot of workload, and hence, health workers were unwilling to use the system for reporting cases. The use of COVID-19 antigen RDTs reduced the workload for reporting, as only positive cases were recommended for reporting. There was therefore more acceptance by health workers in using the system.

**Figure 3:**
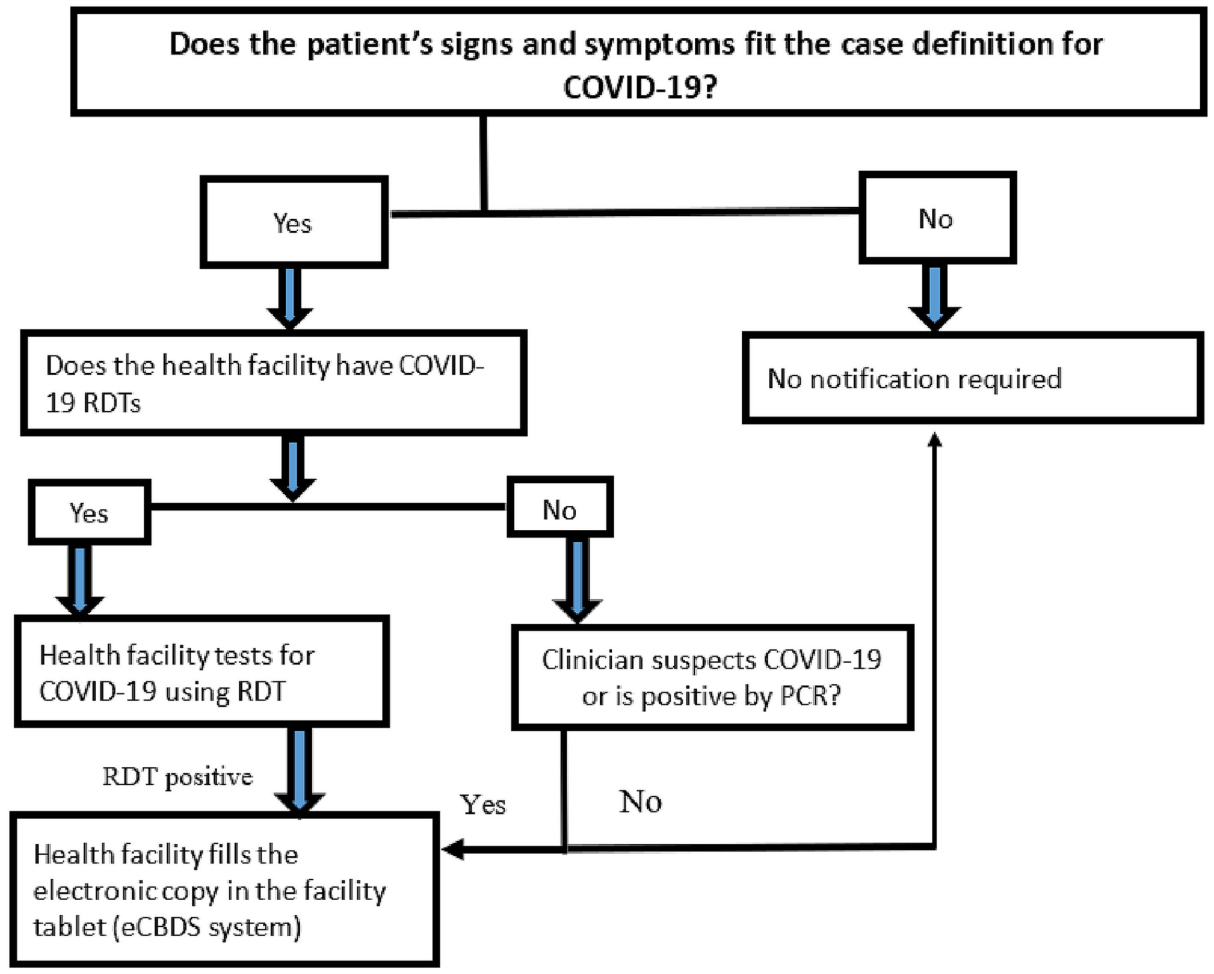
Procedure for notifying a case through the electronic case-based surveillance system.

Once a positive COVID-19 case was detected at the health facility, the case was registered in the eCBDS by the facility surveillance focal person using an Android app (Figure 3). Registration captured basic information such as demographic, geographic and clinical information. The system also captures key indicators to monitor the effectiveness of the outbreak response. Among this include time from symptom onset to detection, time from outbreak detection to notification, time from notification to investigation and time from sample collection to result feedback. Additionally, the system included validation rules to ensure data quality. Once the case was registered, a system generated unique identifier was used to track the case. Upon successful registration, a notification was sent by email or short message service (SMS) to the district surveillance officer for further follow up and investigation. There is also a laboratory module that allows recording of laboratory information including results which are accessible by the health facility. This data collection and reporting process was applicable to all other diseases reported using the system.

### 2.7 Data collection and analysis

Training data was collected through pre and posttest. Data on health facility reporting for COVID-19 and other diseases was collected through the eCBDS system. Quantitative data was downloaded and analyzed using Microsoft Excel and Epi info. The quantitative data was supplemented by qualitative data that was collected from selected about 20 key informants through face-to-face interviews using a semi structured questionnaire. The key informants were drawn from 10 health facilities in Western Area Urban district and also from national and district surveillance officers.

## 3 Results

### 3.1 Quantity and quality of training

**Figure 4:**
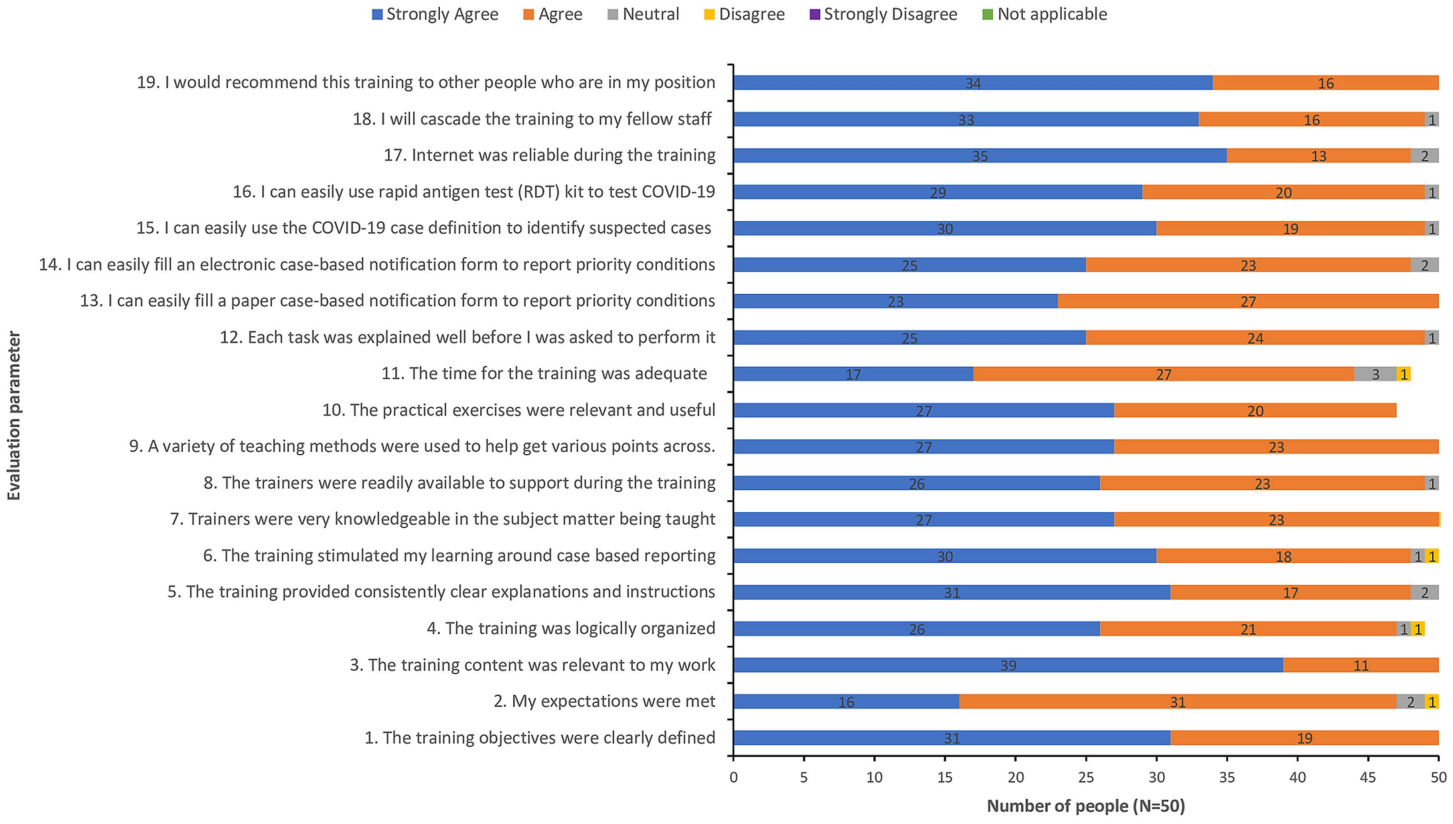
Post-training evaluation feedback from a sample of 50 participants.

The total number of health workers directly trained on the use of COVID-19 RDTs and eCBDS reporting for COVID-19 and other diseases was 607. Majority (350/607 or 58%) of these health workers came from Western Area Urban district, where 60% of COVID-19 cases came from. The remaining health workers came from the remaining 15 districts. The training was generally successful as demonstrated by the post-training evaluation feedback report, which showed that the training was well organized and that participants felt that they were competent enough to use the eCBDS system as well as confirm cases of COVID-19 using RDTs as shown in Figure 4.

### 3.2 Health facilities reporting in eCBDS

The number of health facilities trained nationally was 155/1423 (11%). Though few, the selected health facilities were considered the most important for COVID-19 reporting as these were the sites where most COVID-19 cases were being reported at the time. The training was expected to be catalytic and complementary to the work of other stakeholders and development partners. It was expected that the trained district-level TOTs and health workers would mentor other health workers in the facilities not included in the study. Therefore, the outcome was expected to be significant in ensuring electronic case-based reporting for confirmed COVID-19 cases and other diseases. This was indeed confirmed by the trained health workers (key informants) at the health facilities who indicated that they had mentored one to five more health workers per health facility.

**Figure 5:**
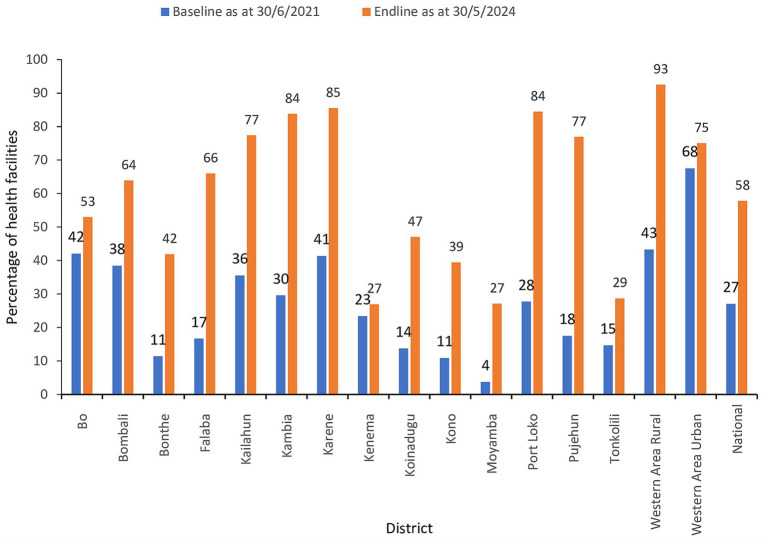
Percentage of health facilities that had reported at least one case of a notifiable disease in eCBDS.

The number of health facilities that had ever reported a case of a notifiable disease through the eCBDS was 385/1,423 (27%) as of 30th June 2021 which was considered as the baseline. As of 30th May 2024 (endline), the number of health facilities had increased to 58% although the increase varied by district as shown in Figure 5. All districts had an increased number of facilities reporting with Pujehun, Port Loko, Kambia, Falaba and Western Area Rural showing the greatest improvement. Before the training, Western Area Urban district, had a relatively higher proportion of health facilities that had reported at baseline (68%). This was because the district was the epicenter of the COVID-19 outbreak and hence many cases had been reported through the system but mostly through the support of District Surveillance Officers. About 41% of the health facilities that had experienced use of eCBDS in Western Area Urban district had less than five cases reported in the system and hence still needed the training support to master the use of the system.

**Figure 6:**
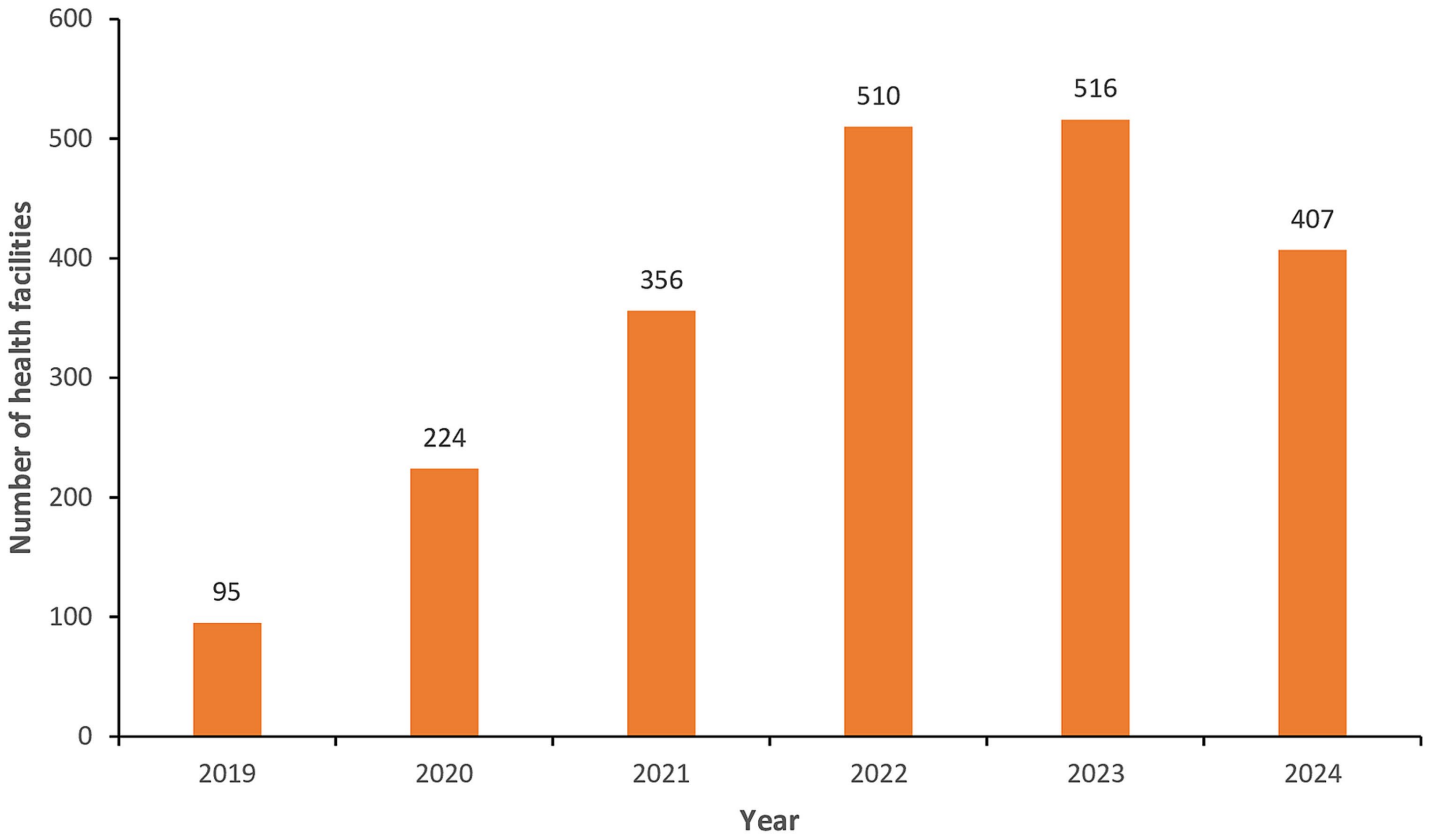
Number of health facilities that reported at least one notifiable case in eCBDS, Jan 2019–Dec 2024.

The eCBDS system started capturing data from 2019 when at least 95 health facilities reported at least one case in that year. The COVID-19 pandemic that started in the country from March 2020 provided an opportunity for health facilities to use the eCBDS system and a gradual increase in health facilities using the system was noted from 2020 to 2023 (Figure 6). Our training on use of eCBDS was conducted from November 2021 to May 2022 and it is likely that this partly contributed to the increased number of health facilities that used the eCBDS system in 2021 to 2023. Due to a reduction in global cases, the World Health Organization declared in May 2023 that COVID-19 was no longer a public health emergency of international concern and this is the reason fewer health facilities reported in 2024.

### 3.3 Diseases reported through eCBDS

COVID-19 was used as the proxy disease for the training of health workers, as it was the most common disease at the time. The eCBDS system captured suspected cases of various notifiable diseases that have been prioritized in the country. The total number of cases (all diseases) reported in eCBDS from 2019 to 30th May 2024 was 54,794. Of the reported cases, 44,908(82%) were suspected COVID-19 cases, of which about 17% were confirmed positive. The top 10 diseases/conditions that were captured in the system includes, suspected COVID-19 (44,908), Measles (2981), dog bites (1842), snake bites (917), viral hemorrhagic fevers (1105), acute flaccid paralysis (709), bacterial meningitis (449), suspected yellow fever (442), bloody diarrhea (283) and Adverse event following immunization (196).

**Figure 7:**
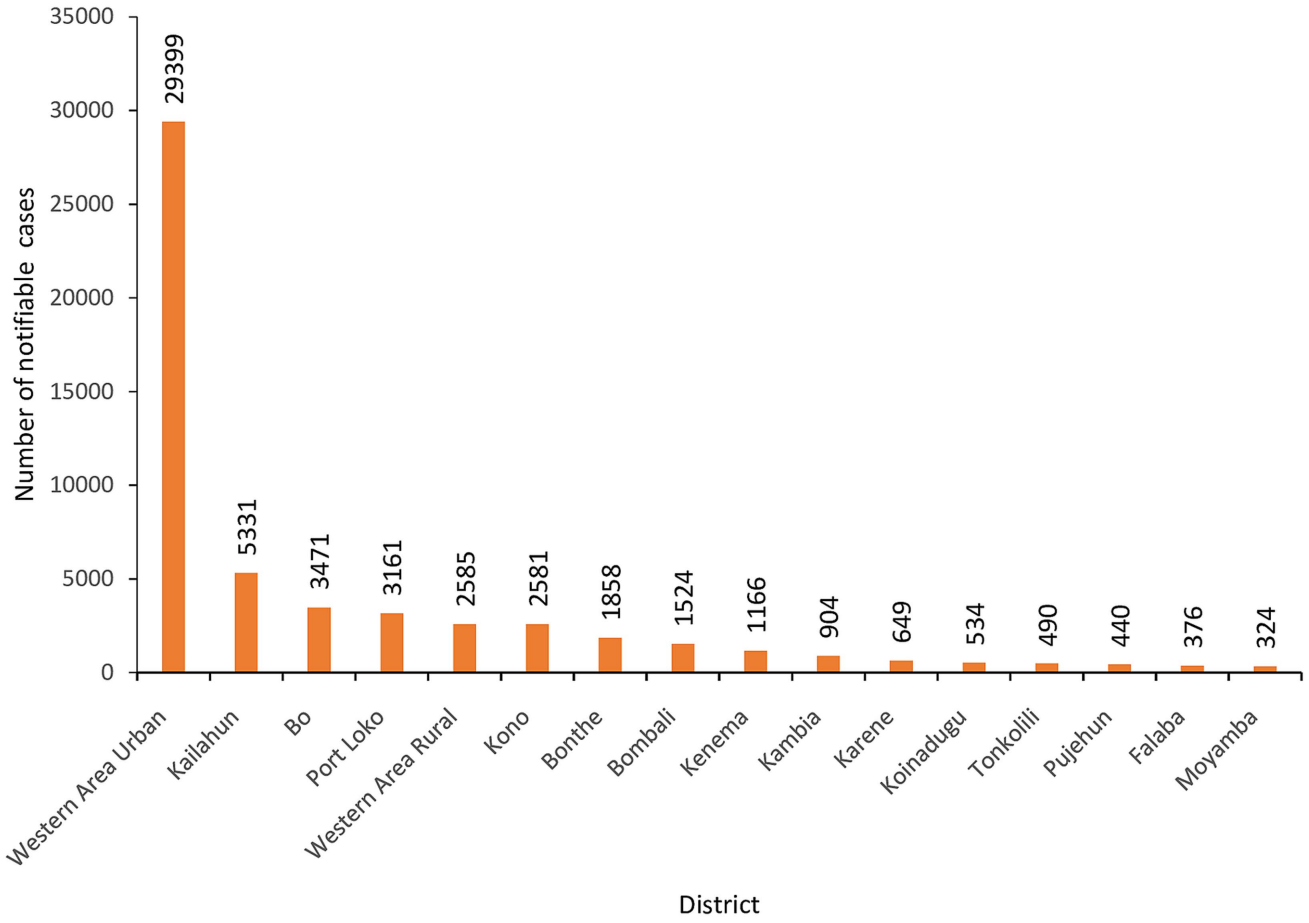
Cumulative number of notifiable disease cases reported in eCBDS by district, as of 30th May 2024 (*N* = 54,794).

The training prioritized Western Area Urban district, the capital city, as it was the epicenter of the COVID-19 in the country and reported the highest number of cases. Cumulatively, the district had 29,399 (54%) cases of all notifiable diseases/conditions that were reported in eCBDS as of 30 May 2024 (Figure 7). Majority (89%) of these cases in the district were suspected COVID-19.

### 3.4 Notification and response timelines

The eCBDS in the country allows tracking of timeliness for outbreak notification and investigations. Health facilities are expected to notify the district health management team within 24 h whenever they encounter a notifiable case. The district health management team is then required to conduct investigations within 48 h after notification. Using COVID-19 as an example, we noted that timeliness of notification and investigation was very good throughout the pandemic period. Before July 2021(baseline), 97.3% of suspected COVID-19 cases were notified to the district by the health facilities within 24 h and this improved slightly to 98.1% afterwards. Timeliness of investigations as measured by when a laboratory sample was taken after notification was also monitored. For the cases that had samples taken, we observed that laboratory investigation (sample collection) within 24 h of notification improved markedly from 91.6 to 98.2% before and after July 2021.

## 4 Discussion

The capacity building project for electronic case-based reporting that was implemented in Sierra Leone in 2021/2022 was catalytic in scaling up use of electronic case-based disease surveillance (eCBDS) for COVID-19 as well as other notifiable diseases. This is demonstrated by the high number of suspected COVID-19 cases reported through eCBDS as well as the doubling of health facilities that had used the eCBDS system between July 2021 and May 2024. While this was commendable improvement, it was noted that there were still many health facilities that did not get a chance to report despite having the reporting devices (tablets) that were fully installed with the eCBDS reporting app. The reasons for not using the system were varied but were mostly due to unavailability of notifiable cases as not all health facilities detected COVID-19 cases.

While electronic aggregate reporting of cases is common in most African countries due to the availability of DHIS2, electronic case-based reporting had not picked up before the COVID-19 pandemic era. Due to limited resources and workload, case-based reporting was mainly limited to use for diseases earmarked for elimination and eradication such as measles and polio (17). Electronic case-based reporting has also been used for various diseases for case investigations, contact tracing and quarantine management (18, 19). However, the COVID-19 pandemic provided unique opportunity to many countries to build the capacity for electronic case-based reporting.

Use of digital systems was one of the innovative strategies to strengthen health system resilience during the COVID-19 pandemic although the choice of which platforms to use for COVID-19 reporting varied by country (20). For sustainability purpose, Sierra Leone opted to leverage on the existing eCBDS platform which is built on an open source DHIS2 tracker and was useful for outbreak investigations, contact tracing and vaccination data management. Many other countries also leveraged on the existing DHIS2 platform for COVID-19 case based reporting (4). Some countries also used Go.Data tool which is an outbreak response tool developed by the World Health Organization (WHO) in collaboration with the Global Outbreak Alert and Response Network (21). In United States, most Public Health Agencies adopted existing tools with the most common ones being the National Electronic Disease Surveillance System Base System (NBS), Sara Alert, REDCap, and Maven (22). Kenya also used an event-based surveillance tool known as m-dharura to complement case-based surveillance to report COVID-19 related signals (23).

Previous studies have shown that early intervention with the use of digital tools had a strong correlation with the successful containment of COVID-19 (24). It is also evident that many countries used digital tools for reporting, which contributed to improving response timelines. Our study additionally shows that notification of COVID-19 cases to the next level (district and national level) was very high throughout the COVID-19 period (before and after training) probably because of the attention given to the disease as a pandemic (more than 97% of cases were notified within 24 h of detection). Compared to the baseline data, we noted that the timeliness of laboratory investigations (sample collection) within 24 h improved markedly (from 91.6 to 98.2%). This improvement may be due to several factors, but capacity building, digitization of the notifications and availability of RDTs played a crucial role in this change.

Most of the health workers interviewed as key informants said that the eCBDS app was easy and friendly to use in reporting cases in Sierra Leone. However, they noted some challenges encountered in the use of the electronic platform. The most commonly encountered challenges include the lack of data bundles and electricity for charging the electronic devices. These challenges were however addressed with the support of partners by providing data bundles and solar power banks. In addition, unlike case-based reporting for other diseases like Measles, which are relatively few, case-based reporting for the many COVID-19 cases was tedious and time-consuming and therefore required increased human resources. Unfortunately, there were only a few health workers in most peripheral health facilities, and COVID-19 reporting was therefore considered extra work and a burden to the health workers. This limited COVID-19 case-based reporting.

To address the challenge of extra workload that was brought about by the COVID-19 case-based reporting, we introduced the use of COVID-19 antigen RDTs (25). This meant that only positive cases were to be notified through the eCBDS system and this greatly reduced the workload. The project promoted use of COVID-19 antigen RDTs by supplying them to all health facilities where health workers were trained. Health workers were therefore only required to report a case in the system if it was positive by RDTs or by polymerase chain reaction (PCR). This improved acceptance in use of the system as workload was reduced. For those facilities, that did not have the RDTs, health workers were still encouraged to report suspected cases if they felt strongly that they met the case definition. The use of RDTs could have contributed to the improved timeliness of laboratory investigation that was noted to have improved markedly after the training.

Several lessons were learnt throughout the process. First, developing eCBDS on existing DHIS2 platform was effective, efficient, and more sustainable as digital infrastructure was already existing at all levels from the health facility up to the national level. Second, COVID-19 promoted use of eCBDS due to the many cases that provided an opportunity for reporting by many health facilities. When COVID-19 cases reduced such as in 2023, we noted that the number of health facilities reporting also reduced compared to 2022. In the absence of COVID-19 cases, the country may therefore want to select another more common disease to ensure that health workers can continue to practice case-based reporting. Third, use of COVID-19 RDTs improved timeliness of laboratory investigation and reduced the burden of case-based reporting as only positive cases required to be notified. Fourth, while health workers expressed satisfaction with class-based trainings for eCBDS, they opined that on-job training and mentorship was more effective in mastering the use of the system and therefore more mentorship visits by the district supervisors should be encouraged.

Due to the many stakeholders who were also contributing to the COVID-19 response, this study had one limitation of inability to directly attribute the improvement in reporting rates to our work in capacity building. However, the study may have contributed greatly to this success.

## 5 Conclusion

A case-based surveillance system is important to public health practice as it allows better understanding of a disease and its risk factors and hence guide appropriate actions for control. In countries where electronic health records (EHR) are largely in use, case-based data is usually easily generated from the systems and has the potential to further increase the breadth, detail, timeliness, and completeness of public health surveillance and thereby provide better data to guide public health interventions. In the absence of electronic health records, implementing electronic case-based surveillance system is usually challenging due to technological, financial, and human resource challenges.

COVID-19 pandemic provided a unique opportunity for the country to scale up electronic case-based disease surveillance reporting. This was not only because of the many cases available for reporting (82 % of all cases ever reported through eCBDS were COVID-19), but also because the traditional technological, financial, and human resource challenges were often addressed. With financial resources from Government and partners, the country was able to scale up electronic case-based reporting for COVID-19 and other diseases through capacity building of health workers. However, more still need to be done to ensure that all facilities are reporting through the eCBDS whenever they detect a notifiable case.

## Data Availability

The original contributions presented in the study are included in the article/supplementary material, further inquiries can be directed to the corresponding author.
